# The Association Between Heterosexual anal Intercourse and HIV Acquisition in Three Prospective Cohorts of Women

**DOI:** 10.1007/s10461-023-04115-y

**Published:** 2023-07-01

**Authors:** Romain Silhol, Ashley Nordsletten, Mathieu Maheu-Giroux, Jocelyn Elmes, Roisin Staunton, Branwen Owen, Barbara Shacklett, Ian McGowan, Kailazarid Gomez Feliciano, Ariane van der Straten, Leigh Anne Eller, Merlin Robb, Jeanne Marrazzo, Dobromir Dimitrov, Marie-Claude Boily

**Affiliations:** 1 MRC Centre for Global Infectious Disease Analysis, Department of Infectious Disease Epidemiology, School of Public Health, Imperial College London, St Mary’s Hospital, 2 Norfolk Place, London, W2 1PG UK; 2 HIV Prevention Trials Network Modelling Centre, Imperial College London, London, UK; 3 Department of Psychiatry, University of Michigan, Ann Arbor, MI USA; 4 Department of Epidemiology and Biostatistics, School of Population and Global Health, McGill University, Montréal, Canada; 5 Department of Medical Microbiology and Immunology, University of California, Davis, Davis, CA USA; 6 School of Medicine, University of Pittsburgh, Pittsburgh, PA USA; 7 FHI 360, Durham, NC USA; 8 Center for AIDS Prevention studies, University of California, San Francisco, CA USA; 9 ASTRA Consulting, Kensington, CA USA; 10 Military HIV Research Program, Walter Reed Army Institute of Research, Silver Spring, MD USA; 11 Henry M. Jackson Foundation for the Advancement of Military Medicine, Bethesda, MD USA; 12 Department of Medicine, University of Alabama at Birmingham, Birmingham, AL USA; 13 Vaccine and Infectious Disease Division, Fred Hutchinson Cancer Center, Seattle, WA USA

**Keywords:** Anal intercourse, Sexual behavior, Heterosexual, HIV incidence, Women

## Abstract

The extent to which receptive anal intercourse (RAI) increases the HIV acquisition risk of women compared to receptive vaginal intercourse (RVI) is poorly understood. We evaluated RAI practice over time and its association with HIV incidence during three prospective HIV cohorts of women: RV217, MTN-003 (VOICE), and HVTN 907. At baseline, 16% (RV 217), 18% (VOICE) of women reported RAI in the past 3 months and 27% (HVTN 907) in the past 6 months, with RAI declining during follow-up by around 3-fold. HIV incidence in the three cohorts was positively associated with reporting RAI at baseline, albeit not always significantly. The adjusted hazard rate ratios for potential confounders (aHR) were 1.1 (95% Confidence interval: 0.8–1.5) for VOICE and 3.3 (1.6–6.8) for RV 217, whereas the ratio of cumulative HIV incidence by RAI practice was 1.9 (0.6-6.0) for HVTN 907. For VOICE, the estimated magnitude of association increased slightly when using a time-varying RAI exposure definition (aHR = 1.2; 0.9–1.6), and for women reporting RAI at every follow-up survey (aHR = 2.0 (1.3–3.1)), though not for women reporting higher RAI frequency (> 30% acts being RAI vs. no RAI in the past 3 months; aHR = 0.7 (0.4–1.1)). Findings indicated precise estimation of the RAI/HIV association, following multiple RVI/RAI exposures, is sensitive to RAI exposure definition, which remain imperfectly measured. Information on RAI practices, RAI/RVI frequency, and condom use should be more systematically and precisely recorded and reported in studies looking at sexual behaviors and HIV seroconversions; standardized measures would aid comparability across geographies and over time.

**Supplementary Information:**

The online version contains supplementary material available at 10.1007/s10461-023-04115-y.

## Introduction

Systematic reviews of cross-sectional studies have shown that heterosexual anal intercourse (RAI) is prevalent worldwide, with little apparent variation by key demographic characteristics such as age [1–9]. Our understanding of the contribution of RAI to HIV incidence is, however, limited by the variability of RAI measurements and exposure definitions across studies and by the scarcity of longitudinal data that tracks levels and persistence of RAI, and HIV seroconversions over time.

Current evidence suggests that RAI increases HIV risk. The pooled estimate of the per-act probability of HIV acquisition risk during one RAI sex act, from a systematic review of serodiscordant-couple studies, was 1.25% (95%CI 0.55–2.23%) [10], a figure ~ 3–20 times higher than that for one receptive vaginal intercourse (RVI) sex act (0.12%, 95%CI 0.08–0.20%)) [11]. However, the exact magnitude of the increase in HIV risk per RAI sex act for women remains uncertain given the large confidence intervals and because the pooled estimate relies on five studies, including four among men who have sex with men [10].

Mathematical model results suggest that, even infrequent RAI (e.g. assuming 7.5% of all acts are RAI) could account for a substantial fraction of new HIV infections among women (~ 23%) if it increases HIV risk by ~ 1.8-fold (equivalent to assuming a 5-fold increase of the acquisition probability at the per-sex-act level) [4, 12] and could influence the impact of prevention strategies such as vaginal microbicide or oral PrEP, which have varying efficacy by anatomical site [13–15]. Nevertheless, HIV trials and cohort studies alike often give little consideration to the impact of RAI practice on HIV incidence during follow-up.

A recent systematic review of longitudinal HIV studies that included some measure of RAI and HIV incidence estimated that women reporting RAI were, on average, 1.6 times more likely to acquire HIV than women not reporting RAI (with some variation in magnitude by risk population and region) [16]. As expected, this figure was lower than the increase in HIV risk per RAI sex act, since women reporting RAI do not practice it in all their sex acts and differences in per-act risk translates into smaller differences in cumulative incidence risk [7]. However, the review also highlighted limitations which could explain differences across studies and obscure the association, including variable definitions and recall periods for RAI exposures, the near systematic use of non-confidential interview methods potentially resulting in misclassification bias, absence of adjustment for important confounders such as condom use (less than a quarter were adjusted for possible confounders), and lack of accounting for changes in RAI practices during follow-up.

Existing knowledge of RAI practice among women has largely focused on reporting the prevalence of RAI over long recall periods (lifetime, past year) [8], with frequent variation in estimates across studies, likely influenced by reporting biases due to the use of non-confidential interview methods, such as face-to-face interviews and/or challenges with accurate translation of sexual terms [17]. Very few cohorts have considered the prevalence and persistence of RAI over time [18–20]. For example, of 31 systematically-reviewed studies on the practice of heterosexual anal intercourse in South Africa, only one reported RAI prevalence over two different time frames [3]. Further, in a systematic review looking at the increased HIV incidence due to RAI exposure, only a quarter of the estimates accounted for changes in RAI practices over time, which should influence the magnitude of HIV risk cumulatively over the study period [16] and could explain why longitudinal studies among women have estimated a lower increase in HIV acquisition risk due to RAI than per-act estimates which draw on serodiscordant partnerships studies or prospective cohorts of MSM.

To address this knowledge gap and better understand potential sources of variation in estimates of the magnitude of association between RAI and HIV across studies, we used longitudinal data from three recent cohort studies conducted in Africa (MTN-003 (VOICE) trial and RV 217) and in the Caribbean (HVTN 907) [21–23] to (1) examine the level and persistence of RAI practice (e.g. prevalence) among women over the study periods and to (2) assess the relationship RAI and HIV incidence using different RAI exposure definitions. Based on existing knowledge we hypothesized that HIV incidence will be higher among women reporting RAI than not reporting, and that the magnitude of association would be higher for women reporting RAI more consistently (RAI in the past few months before follow-up visits) and/or frequently (percentage of all acts that are RAI).

## Methods

### Description of the Three Longitudinal Studies (VOICE, RV 217, and HVTN 907)

We analyzed data from three recent longitudinal studies. The MTN-003 Vaginal and Oral Interventions to Control the Epidemic (VOICE) trial recruited sexually active women aged 18–40 years (in South Africa, Uganda, and Zimbabwe; N = 5,029) who reported at least one act of RVI in the three months preceding the baseline interview [23]. We used data from all trial intervention and control arms, as none were associated with lower HIV incidence due to low product adherence [24]. The prospective study of acute HIV infections in adults (RV 217) recruited women aged 18–47 years with a higher risk profile (in Uganda and Kenya; N = 1,545) from locations associated with transactional sex (e.g. bars and clubs) [21]. Finally, the prospective cohort study HVTN 907 recruited Caribbean female sex workers (FSW) aged 18–45 years (in Haiti, the Dominican Republic, and Puerto Rico; N = 1,019) who had performed at least one condomless act of RVI or RAI in the last six months [25]. The main characteristics of the studies and participants are summarized in Table 1, with more complete descriptions, including protocols and ethics approvals, available elsewhere [21, 23, 25]. Further ethics approval for our secondary analysis was obtained from Imperial College London (approval # 16IC3667).

**Table 1:** Overview of HVTN 907, VOICE, and RV 217 studies’ characteristics, including a description of key demographic and behavioral participant characteristics

	VOICE (Southern Africa)	RV 217 (East Africa)	HVTN-907 (Caribbean)
**Study overview:**			
Years of data collection	2009–2012	2009–2018	2009–2012
Site	South Africa, Uganda, and Zimbabwe	Uganda and Kenya	Haiti, Dominican Republic, and Puerto Rico
Study type	Randomized trial of oral and gel pre-exposure prophylaxis (PrEP)	Observational study	Observational study
Sample Size (baseline)	N = 5,029	N = 1,545	N = 1,019
Data Method	Audio computer-assisted self-interview (ACASI)	Audio computer-assisted self-interview (ACASI)	Face-to-face interview (FTFI)
Population Type	Females at risk of HIV infection	FSW (90%) & high-risk females	FSW
Recruitment	STI/family planning/postnatal clinics	Community/ street outreach	Street outreach
Age Range	18–40	18–47	18–45
Inclusion criteria	HIV-uninfected and sexually active (RVI in last 3 months)	Exchanged sex and condomless RVI or condomless RAI with ≥ 3 partners or ≥ 1 HIV-positive partners	Exchanged sex plus condomless RVI or condomless RAI in prior 6 months
Follow-up visits (behavioral)	Every 3 months for 3 years	Every 6 months for 2 years	Every 6 months for 18 months
Person-years of follow-up	5,509	1,589	1,119
Injection Drug Use	Excluded	Included	Included
HIV test window and	1 month (rapid test)	10 days (RNA tests)	1 month (normal test)
frequency of HIV testing	Monthly	Twice weekly	Every 6 months
HIV Incidence (seroconversions during follow-up)	306 infections	34 infections	12 infections
**Receptive anal intercourse (RAI) and demographic variables**:			
RAI Variables	RAI in last 3 months Number (No.) of RAI acts in last 3 months	RAI in last 3 months RAI in last 3 months per partner type No. RAI partners/partner type^a^	RAI in last 6 months No. of RAI acts in last week
Condom use during RAI	During last RAI	During last RAI with each partner type^a^	Only the number of condomless RAI in prior week was reported
Age categories	< 25, 25 + years	< 25, 25 + years	< 25, 25 + years
Trial arms	All^b^	NA	NA
Ethnicity	NA	NA	Hispanic/Black/White/Other
Injection Drug Use	NA	IDU history	IDU and non-IDU history
**Other sexual behaviors variables**:			
Sex work	Prior year	Prior 3 months	Prior 6 months
Vaginal intercourse	No. RVI in prior week	No. acts (not reported if RAI or RVI) in prior 3 months, by partner type^a^	Only the number of condomless RVI in prior week was reported
Condom use during RVI	Last RVI in last week	Last intercourse (not reported if RAI or RVI), by partner type^a^	NA (see above)

RAI = receptive anal intercourse; IDU = injecting drug users; NA = not available; PrEP = pre-exposure prophylaxis; STI = sexually transmitted infection; RVI = vaginal intercourse

^a^ Four partner types were defined during the RV217 study: (1) steady partners, (2) casual partners, (3) client (commercial sex partners), and (4) “other” partners (which includes boss/work supervisor, teacher, other authority figure and relative other than spouse, student, employee/work subordinate, or rapist)

^b^ Every trial arms were included for this analysis because none of them were associated with higher HIV incidence [23]

### Study Variables

Behavioral data was collected via *Audio Computer-Assisted Self-Interview* (ACASI) at baseline and every three months (VOICE) or six months (RV 217), or via Face-to-face interviews at baseline and every six months (HVTN 907). History of sex work was reported for the prior year (VOICE), prior 3 months (RV 217), or prior 6 months (HVTN 907), while history of drug injection was recorded at baseline only for RV 217 (ever/never/recently injected) and HVTN 907 (in past 6 months; see Table 1). Information on RAI practice, at baseline and follow-up, was elicited over either the past 3 months (VOICE and RV 217), or the past 6 months (HVTN 907; see Table 1 and Figure S1). Although women enrolled in RV 217 were followed every six months, the study item used a recall period of three months (Figure S1). We therefore assumed participants’ report of RAI practice over the past six months was the same as over the past 3 months. RAI prevalence was defined as the proportion of the study population that reported having practiced RAI over a specific recall period. Information on the number of RAI acts during recall periods was available for VOICE and HVTN 907 participants (Table 1 and S1). RAI fraction was defined as the fraction of total sex acts (RVI plus RAI) that were RAI, only among those that reported this sexual behavior.

In VOICE, condom use at last RVI was reported only by participants who had a sex act in the last week, whereas condom use during RV 217 was reported for the last intercourse (RAI or RVI) by partner type (steady, casual, client); the prevalence of condomless RAI or fraction of condomless sex acts that are RAI in these two studies could not be ascertained because data only covered the last RAI. Information on condom use during sex acts was not collected during HVTN 907.

**Fig. 1:**
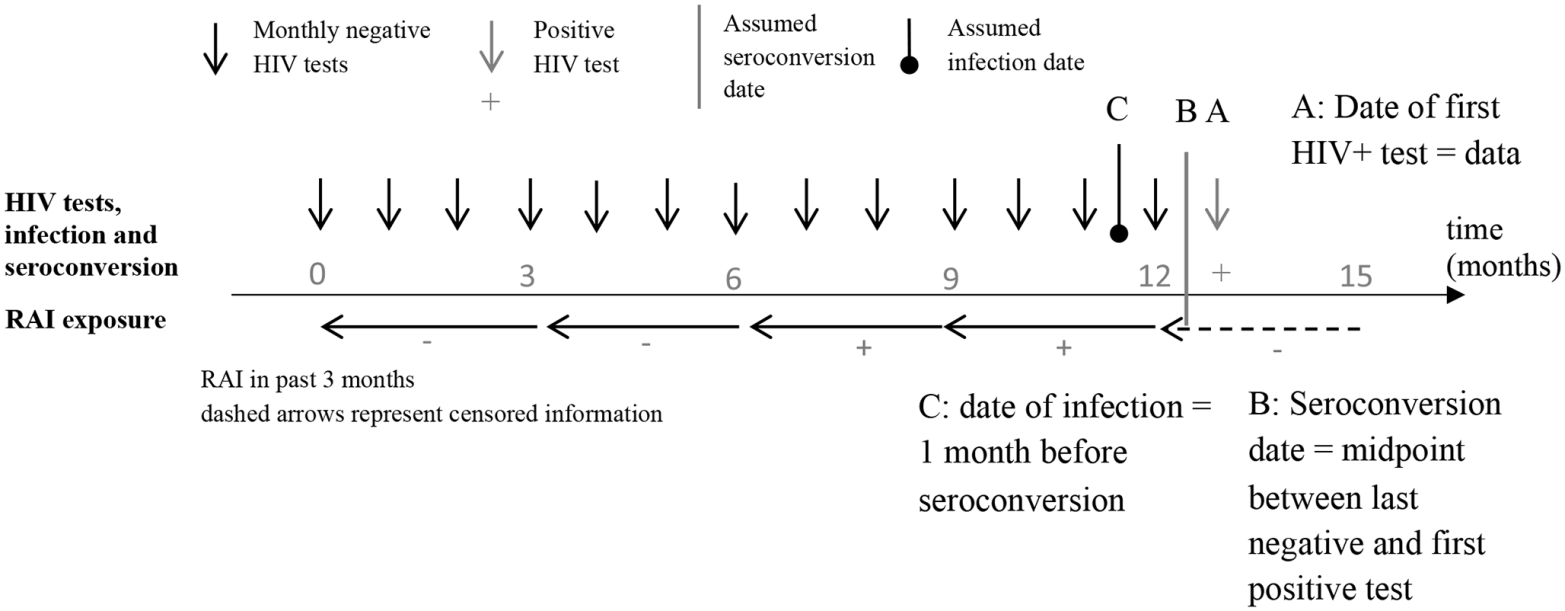
Inferring the date of HIV infection (C) among VOICE participants. In this example, the behavioural data collected after 15 months (dashed arrow) was not used because the covered period was after the estimated infection time. The plausible exposure to receptive anal intercourse (RAI) considered in the analysis is represented by “+”

Incident HIV infections were measured by testing participants once a month in the VOICE trial using two different third-generation rapid tests (positive assays were confirmed by a GS HIV-1 western blot) [23] and every six months in the HVTN 907 study using ELISA tests [25]. HIV-negative women enrolled in RV 217 were tested twice a week using Aptima RNA tests on small-volume blood samples [21]. Because HIV testing protocols varied between studies, dates of HIV infection were inferred in different ways. The seroconversion time was assumed at mid-point between the last and current visit for VOICE and HVTN 907, and HIV infections of participants were assumed to have occurred one month before seroconversion time (Fig. 1 for VOICE). For participants of RV 217, we assumed that HIV infection occurred two weeks before the first positive HIV test to reflect the higher testing frequency and very short detection window of HIV RNA tests.

### Statistical Analyses

Sociodemographic and behavioral differences between women reporting RAI at baseline (hereafter called “*RAI + women*”) and women only reporting RVI in each study at baseline (hereafter called “*RVI-only women*”) were evaluated using Pearson chi-squared and Wilcoxon signed-rank tests. The persistence of RAI practices over time was characterized using four outcomes: (1) cross-sectional RAI prevalence (proportion of women reporting RAI over the previous 3 months (6 months for HVTN)) at baseline and at each subsequent follow-up visit, (2) anytime RAI prevalence (proportion of women that reported RAI at baseline or any of the follow-up visits), (3) the proportion of women reporting RAI for the first time and (4) the proportion of women who stopped practicing RAI during follow-up. Outcome 1 was also stratified by participant socio-demographic characteristics. Outcome 3 was estimated from prevalence at the first follow-up visit, but only among women not reporting RAI at baseline. Outcome 4 was estimated from the prevalence at the first follow-up visit, but only among women reporting RAI at baseline. Total changes in RAI prevalence during the study were measured by comparing RAI prevalence at the first (baseline) and last visit and compared using chi-squared test for linear trend in proportions. Finally, we derived the cross-sectional fractions of all sex acts that were RAI and fractions of RAI and RVI acts involving condoms for VOICE and RV 217 over time.

Because only 12 seroconversions were observed in the HVTN 907 study, the association between RAI practice and HIV incidence was calculated as the ratio of crude cumulative incidences among *RAI +* and *RVI-only women*. Otherwise, the VOICE trial and the RV 217 studies were used for detailed analyses using five different RAI exposure definitions (D1-D5) commonly used in previous analyses [16]. The first one consisted of having reported RAI at baseline (D1; in the past three months for VOICE and RV 217). The second definition reflected RAI persistence during follow-up visits; each woman was classified as having never or ever reported RAI during follow-up (D2). The third RAI exposure definition classified participants as never, sometimes (D3_a_), or consistently (always) (D3_b_) reporting RAI during follow-up (excluding baseline information). The fourth RAI exposure definition was time-varying based on reports of RAI practice at each visit (D4; baseline and follow-up) [26, 27]. The last definition was based on the RAI fraction (for VOICE trial only) and was expressed as the proportion of all sex-acts that were RAI among those reporting RAI during follow-up (D5_a_: 1–30% vs. no RAI; D5_b_: >30% vs. no RAI). The associations between D1-D5 and incident HIV were first estimated using univariate Cox Proportional hazards models. Analyses were performed separately for the different studies due to differences in populations and study designs.

Potential confounders of the RAI-HIV relationships were adjusted for in multivariable analyses using available data from each study. For the VOICE trial, the multivariable model was adjusted for baseline data on age at enrolment (18–25, 25 + years), country (South Africa, Uganda, or Zimbabwe), trial arm (control vs. placebo), sex work (prior year), number of partners (1, 2, 3 + in the past 3 months), and condom use at last vaginal sex. The adjusted model for the RV 217 study included age (18–25, 25 + years), country (Uganda or Kenya), number of partners in the last 3 months (< 10, 10+), history of injecting drug use (never/ever), and condom use at last sex. None of the statistical models could be adjusted for condom use at last RAI because it was highly correlated with condom use at last RVI at the overall and individual level in VOICE (69% vs. 71%, with > 90% of women reporting using a condom at last RAI also reporting using a condom at last RVI) or correlated with condom use at last sex by partner type in RV 217 (e.g. 47% vs. 53% at last sex with a client, with 75% of women reporting using a condom at last RAI with a client also reporting using a condom at last sex with a client), and because condom use at last RAI was only reported by *RAI + women*.

A sensitivity analysis evaluated the impact of gradually introducing an improved version of the ACASI questionnaire during the VOICE trial (including improved translation of terms related to RAI practice) on the persistence of RAI by (1) comparing the cross-sectional RAI prevalence for each of the ACASI questionnaire version, and (2) re-estimating of the association between RAI exposure definitions and HIV incidence only among VOICE participants having used the improved questionnaire from baseline (23% of the total population). All statistical analyses were performed using the R software (version 3.5.1) [28].

## Results

### Prevalence, Frequency, and Persistence of RAI Practice in the Three Longitudinal Studies

**Fig. 2:**
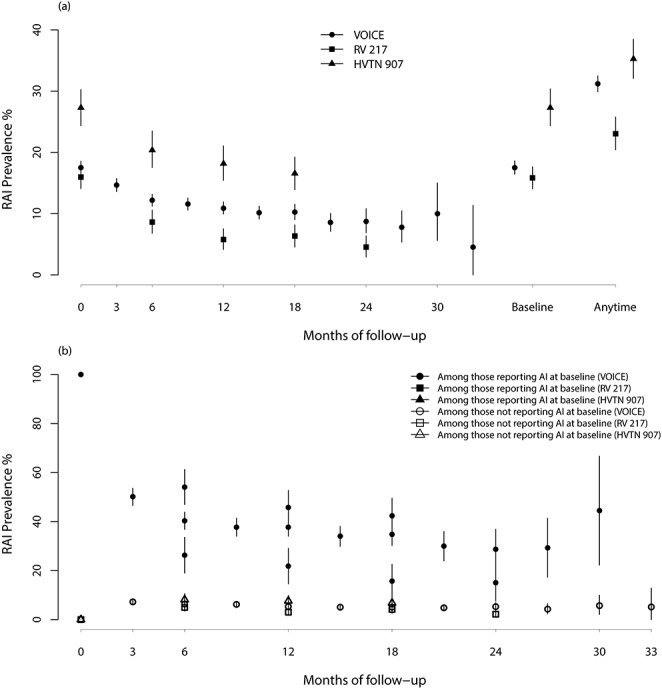
(a) Cross-sectional prevalence of receptive anal intercourse (RAI) at baseline and during follow-up for the three longitudinal studies under consideration (VOICE, RV 217, and HVTN 907). On the right, baseline RAI prevalence (in the past 3 months for VOICE and RV 217, 6 months for HVTN 907), and anytime RAI prevalence (including baseline, correspnding to 3 years, 2 years, and 18 months of follow-up for VOICE, RV 217, and HVTN 907, respectively). (b) Cross-sectional RAI prevalence among women that reported RAI at baseline (filled circles, squares and triangles). The same, but calculated only among women that did not report RAI at baseline (circles, squares and triangles). Error bars represent 95% confidence interavals of estimate

The proportion of women reporting RAI over the last three months at baseline was 17.5% (95% confidence interval (95%CI): 16.5–18.6%) among VOICE participants and 16.0% (14.2–17.9%) among RV 217 participants (Fig. 2). For HVTN 907, prevalence of RAI over the past six months was 27.3% (24.4–30.2%). In VOICE, higher proportions of *RAI + women* lived in South Africa and were in the youngest age group, compared to RVI-only women (Table S2a). Women reporting RAI in RV 217 reported more frequent history of injecting drugs, more sexual partners, and less frequent condom use than *RVI-only women* (Table S2b). In contrast, higher proportions of *RAI + women* in HVTN 907 study were older than 25 years and reported having fewer clients compared to *RVI-only women* (Table S2c).

The anytime RAI prevalence at baseline and during follow-up was 31.1% (29.9–32.5%) for VOICE (over 3 years of follow-up), 23.1% (20.3–25.9%) for RV 217 (over 2 years), and 35.3% (32.1–38.4%) for HVTN 907 (over 18 months, Fig. 2b). RAI prevalence at follow-up visits significantly declined over time in all three studies (Chi-square test for trend: p-value < 0.001 for VOICE, RV 217, and HVTN 907) (Fig. 2a). RAI prevalence (past 3 months) decreased to 4.5% compared to baseline (i.e. by 74%) after 33 months of follow-up in VOICE, to 4.5% (by 72%) after 2 years of follow-up in RV 217, and to 16.6% (by 39%) after 18 months of follow-up in HVTN 907. The decline in reported RAI practice was most pronounced between baseline and first follow-up, with 50% of women in the VOICE sample who were *RAI +* at baseline no longer reporting RAI at the three-month follow-up (Fig. 2b). The decline was even steeper in RV 217, with 74% stopping after 6 months. In HVTN 907, the reductions were 46% between baseline and first follow-up – also occurring at six months. The RAI prevalence among these women continued to decrease after this initial follow-up visit, a difference not attributable to differential loss to follow-up among women reporting RAI. Results of the persistence of RAI practice, stratified by socio-demographic characteristics, are presented as supplementary material (Figures S2 to S6). Briefly, the decline in cross-sectional RAI prevalence across the 3 studies was largest for Kenyan participants of RV 217, declining from 14.1% (11.8–16.2%) at baseline, to 1.5% (0.5–2.8%) after two years of follow-up (Figure S2).

Most participants did not practice RAI at any time during follow-up (74.8% VOICE, 86.8% RV 217, and 72.8% for HVTN 907) (Figure S7), with 21.9% (VOICE), 11.1% (RV 217), and 16.3% (HVTN907) of participants, respectively, reporting RAI at least once during follow-up. Very few participants reported practicing RAI at all follow-up visits (3.3% VOICE, 2.1% RV 217, and 10.9% for HVTN 907). Among women ever-practicing RAI (25.2% VOICE, 13.2% RV 217, and 27.2% for HVTN 907), RAI was most often reported at only one follow-up visit.

Importantly, 7.2% (6.4–8.1), 5.0% (3.4–10.6), and 8.1% (5.9–10.6) of the women who did not report RAI at baseline in VOICE, RV 217 and HVTN 907 reported initiating RAI at the first follow-up visit (3, 6, 6 months after baseline, respectively) (Fig. 2b). *RAI + women* who ceased to report RAI at first follow-up were more likely to reside in Zimbabwe (VOICE), Kenya (RV 217), and Haiti (HVTN 907) (Figures S4a-6a). Conversely, being aged under 25 years (VOICE), or residing in either Uganda (RV 217) or Puerto Rico (HVTN), was most associated with reporting initiating RAI at first follow-up visit among *RVI-only women* (Figures S4b-S6b).

**Fig. 3:**
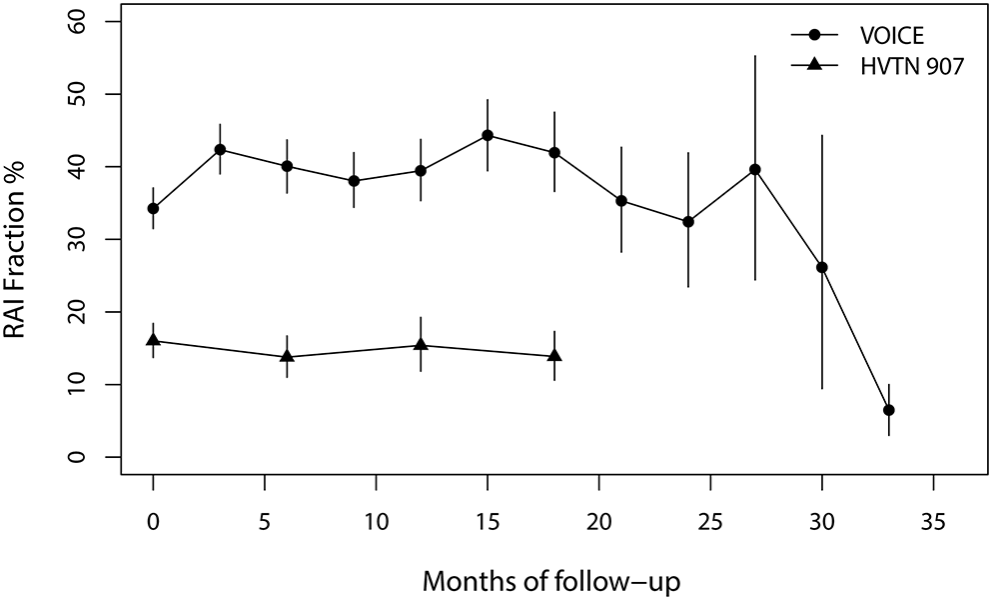
RAI fraction during VOICE (dots) and HVTN 907 (triangles): fraction of sex acts that are receptive anal intercourse (RAI) among all sex acts, calculated among participants who report recent history of RAI at study visit. Error bars represent 95% confidence interval of estimate

At baseline, the fraction of RAI (only calculated among those who reported RAI acts) was 34.2% (31.6–37.1) in VOICE and 16.0% (13.7–18.5) in HVTN 907. These proportions remained stable after initial HIV/STI risk reduction counselling and during follow-up in both studies (Fig. 3 and S8), whilst the fraction of condom use during RVI and RAI decreased slightly over time (Figure S9).

### Incident HIV and RAI Association for Different RAI Exposure Definitions

Overall, we found positive associations between the different RAI exposure definitions and incident HIV across studies, albeit sometimes accompanied by wide confidence intervals (Table 2). Estimates of the magnitude of association with HIV during VOICE and RV 217 varied by definition of RAI exposure. For VOICE, reporting RAI in the three months before baseline was not associated with higher HIV incidence during the trial (aHR = 1.1; 0.8–1.5), however always reporting RAI during follow-up visits was (aHR = 2.0; 1.3–3.1 for D3_b_). The unadjusted time-varying definition (D4) yielded a slightly higher point-estimate of association than D1 (1.2 vs. 1.1), though, contrary to our hypothesis, the reported fraction of acts that were RAI (D5_a,b_) was not associated with HIV incidence. By contrast, and consistent with our hypothesis, in RV 217, reporting RAI in the three months before baseline was associated with a much higher HIV incidence (aHR = 3.3; 1.6–6.8). However, due to low incidence (22 infections) among individuals completing at least one round of follow-up questionnaire, we were not able to calculate adjusted hazard ratio (aHR) for D2-D4, nor use time-varying RAI variables, in this cohort. The association between reporting RAI at any time (D2) or consistently (D3_b_) during follow-up and HIV incidence were not statistically significant despite relatively high HR point-estimates (HR = 1.7; 0.4–7.2 and 2.6; 0.3–19.2 for D2 and D3_b_, respectively). Finally, the RAI HIV cumulative incidence ratio for HVTN 907 was 1.9 (0.6-6.0), based on 12 incident cases (5 among RAI+, 7 among RVI-only women, not shown).

**Table 2:** Epidemiological data used of the longitudinal analysis, and results of statistical associations between receptive anal intercourse (RAI) exposures and HIV infection during the studies, hazard ratios (HR) and adjusted hazard ratios (aHR) point estimates and 95% confidence intervals

	VOICE (Southern Africa)		RV 217 (Eastern Africa)	
**Number of HIV seroconversions**				
Among all participants	312		34	
Among participants completing at least one round of follow-up	306		22	
**Definitions of receptive anal intercourse (RAI) exposure**	HR (95%CI)	aHR (95%CI)^a^	HR (95%CI)	aHR (95%CI)^b^
**D1: RAI at baseline**				
No RAI	1^d^	1	1	1
RAI	1.23 (0.93–1.64)	1.10 (0.82–1.48)	3.26 (1.63–6.5)	3.33 (1.64–6.79)
**D2: RAI during follow-up (excludes baseline information)**				
Never RAI	1	1	1	NA
Ever RAI	0.91 (0.69–1.21)	0.80 (0.60–1.07)	1.68 (0.39–7.18)	NA
**D3**_**a,b**_: **RAI consistency during follow-up (excludes baseline information)**				
Never RAI	1	1	1	NA
Sometimes RAI	0.66 (0.47–0.93)	0.59 (0.41–0.83)	0.34 (0.04–2.51)	NA
Always RAI	2.29 (1.51–3.47)	2.00 (1.29–3.12)	2.57 (0.34–19.17)	NA
**D4: Time-varying RAI exposure** ^**c**^				
No RAI	1	1	NA	NA
RAI reported	1.33 (1.02–1.75)	1.18 (0.88–1.57)	NA	NA
**D5**_**a,b**_: **RAI frequency (fraction of all sex acts that are RAI)**				
No RAI	1	1	NA	NA
1–30%	0.98 (0.71–1.35)	0.86 (0.61–1.20)	NA	NA
>30%	0.81 (0.50–1.29)	0.69 (0.42–1.14)	NA	NA

RAI = anal intercourse; HR = Hazard ratio; aHR = adjusted Hazard ratio; 95%CI = 95% confidence interval, NA = not estimated due to small numbers of seroconversions during the study

^a^ VOICE model adjusted for baseline data on age (18–25, 25 + years), country (South Africa, Uganda, or Zimbabwe), trial arm (control vs. placebo), sex work (prior year), number of partners (1, 2, 3 + in the past 3 months), and condom use at last vaginal sex

^b^ RV 217 model adjusted for age (18–25, 25 + years), country (Uganda or Kenya), number of partners in the last 3 months (< 10, 10+), history of injecting drug use (never/ever), and condom use at last sex (any type of intercourse). The later was defined as condom use at last sex with client, or with a casual partner if condom use with last client was not reported

^c^ calculated using data from each study visit

^d^ referent

### Sensitivity Analysis to the RAI Questionnaire (VOICE)

The RAI prevalence at baseline and during the first year of follow-up was approximately only 1% point lower among VOICE participants using the second questionnaire version compared to the first version (Figure S10). However, the estimated magnitude of association between the different RAI exposure definitions and HIV incidence was similar for the analysis conducted only among study participants using the second questionnaire (23% of participants) than the whole sample (Figure S11).

## Discussion

Our study shows that RAI was commonly reported, but not necessarily practiced regularly during follow up, by women recruited in three different HIV longitudinal studies. RAI practice declined markedly during study periods, but not the frequency of RAI among those reporting RAI. Despite its well-established, heightened per-act probability of HIV acquisition [10], the association between RAI practice and incident HIV infection was not always positive and varied across exposure definitions, settings and studies, highlighting persisting difficulties in precisely measuring unprotected RAI, a relatively infrequent and sensitive behaviour. Consistent with our hypothesis, higher estimates of the magnitude of the RAI and HIV association in VOICE were usually observed when using more precise exposure definitions, such as time-varying RAI exposure or ‘always practicing’ RAI, although the fraction of acts that are RAI was not associated with HIV incidence during VOICE, which departed from our hypothesis.

The prevalence of RAI among women at baseline was consistently higher than 15% across the three studies, and approximately 2-fold higher among HVTN 907 FSW participants (in the Caribbean), which is consistent with findings from a recent systematic review and meta-analysis of the prevalence of RAI among FSW [29], which was found to be around 15–20% among FSW in Africa compared to 20–28% in the Americas [29]. Furthermore, a survey among at-risk women living in 20 U.S. settings also found a 2-fold higher prevalence of RAI in the past year in San Juan (Puerto Rico) compared to other U.S. cities [30], yielding a much higher estimated relative contribution of RAI to new HIV infections in this population than on average over the whole sample (57% vs. 41%). Reporting of RAI by women at higher risk of HIV infection is known to be influenced by contextual factors such as violence and substance use, which vary across settings and populations [20]. Reported RAI consistently declined over follow-up, particularly at first follow-up visit, during all studies. Since the cohorts recruited women at high risk of infection, this may be partly explained by regression to the mean [31], HIV/STI risk-reduction counselling of study participants which took place at every study visit in all studies [23], or increased desirability bias (which may have been influenced by regular counselling and may result in misclassification bias). Interestingly, the decline in RAI contrasts with the reported use of condoms during RVI and RAI during VOICE, which slightly declined over time and may have been partly influenced by beliefs of product efficacy among participants, despite counselling and condom distribution (Figure S9), whilst the participants’ reported number of partners (over the past 3 months) remained stable over time (Figure S12). Our analysis also showed that, although few individuals continued practicing RAI throughout the studies, a non-negligible number (around 8%) reported initiation of RAI at the first follow-up visit (Fig. 2b), despite HIV/STI risk reduction counselling. When available (VOICE, HVTN 907), RAI fraction appeared to remain stable over time at substantial levels among women practicing RAI, and higher in VOICE (at risk women) than in HVTN 907 (FSW), which could explain why the estimated risk of acquiring HIV due to RAI among women consistently practicing RAI over time was high.

In VOICE, the only study which provided sufficient information to conduct all adjusted statistical analysis, the estimates of the magnitude of association were larger for exposure definitions that measured more precise (time-varying) and more consistent RAI exposures, as hypothesized, albeit not necessarily statistically significant. The opposite was found for RV 217 where, contrary to our hypothesis, reporting RAI at baseline was more strongly associated with HIV incidence than when using follow-up data to define RAI exposure. Our estimates are in line with pooled African estimates from the Stannah at al. review, where the crude and adjusted measures of association were 1.2 (0.9 − 1.5) and 2.3 (0.8 − 6.4), respectively, based on 13 studies [16]. In the review, more precise definitions of RAI exposure were also not associated with higher increase in HIV incidence. The lower magnitude of the association during VOICE compared to RV 217 and HVTN may be partly due to the higher incidence of HIV during the study (when risky behaviors are not needed to lead to HIV infection because of higher prevalence among partners or other risk factors among women, their association with HIV incidence is diminished), as well as less precise HIV testing algorithm compared to RV 217.

Limitations to our analysis are primarily due to the low HIV incidence during the RV 217 and HVTN 907 observational incidence studies and limitations on the RAI data and condom use during RAI and RVI. Only 12 incident infections occurred during the HVTN 907 study, and if 34 infections occurred in the Ugandan and Tanzanian RV 217 study sites, only 22 of them occurred among individuals reporting data during follow-up, which reduced our ability to assess the effect of RAI practice since RV 217 and HVTN 907 data could not be combined due to large differences in behavior and HIV measurements. The reliability of the estimated dates of HIV infection depended on the frequency and type of HIV tests used, which were rapid tests used monthly and every 6 months in the case of VOICE and HVTN, respectively, whereas RV 217 used RNA tests twice a week, minimizing the risk of misclassification bias.

Despite efforts and improvements in the methods of data collection, RAI may still be not accurately reported by participants [17]. This might be especially true here since the meaning of RAI is particularly ambiguous in several Southern Africa local languages [17, 32]. The VOICE and RV 217 studies relied on ACASI techniques that may yield higher RAI prevalence estimates, for example due to reduced social desirability biases [3, 33], but this technique does not allow the interviewer to ensure respondents have understood the question well. However, in our sensitivity analysis, more accurate translation of RAI practice questions in local languages on the ACASI questionnaire [23] and reduced possibility of misclassification biases did not result in higher estimates of the RAI prevalence and magnitude of HIV association. The reported fraction of acts that were RAI was not associated with HIV incidence during VOICE, which could be partly due to the inconsistency in the recall periods used to measure RAI and RVI; the number of RVI was reported over only one week compared to 3 months for RAI, leading to significant number of women not reporting RVI but reporting RAI, potentially overestimating the RAI fraction. Misclassification bias was more likely in the RV 217 analysis since the recall period for the question on RAI practice was shorter (3 months) than the time period (6 months) between two behavioral assessments. Finally, condom use during RAI was asked at last sex during surveys, which did not allow us to fully control for it in our statistical models.

Our study has several strengths. Our analysis benefitted from longitudinal data from different studies and contexts, rarely fully described in the literature [19]. Although our analysis improves on estimates of association from cross sectional studies, which are more likely to lead to reverse causation (past HIV infection could explain changes in behaviors), it also highlighted persistent challenges in estimating RAI and its associated HIV risk. Our analyses of the HIV incidence were adjusted on several important cofactors such as number of partners or condom use, and other higher risk practices such as sex work and injecting drugs, leading to more reliable estimates of association. Condom use was available at last sex (or RVI), but also at last RAI specifically for VOICE and RV 217. In VOICE, the levels of condom use at last RVI and last RAI were similar (Figure S9), whilst during RV 217 condom use (reported at last intercourse) was less frequent among *RAI + women* compared to *RVI-only women* (which was accounted for by our statistical models), but condom use by *RAI + women* during RAI was similar to during RVI (Table S1). However, available data did not allow control for the HIV status of the participants’ male partners which was not known. It is possible that the lack of association between RAI and HIV incidence could be due to residual confounding due to unmeasured demographic characteristics and HIV status (including viral load suppression) of the clients of the study participants being able to afford paying for RAI (in one study with African sex workers, charges for RAI were greater than for RVI [12]). Importantly, none of the studies recorded where ejaculation occurred during RAI and RVI, a factor influencing HIV acquisition risk. Estimates suggest that per-act HIV acquisition risk is ~ 3-fold higher when ejaculation occurs in the rectum or vagina, compared to withdrawal [34, 35]). Overall, the modest magnitude of associations estimated during our study was slightly lower than hypothesized based on the large differences in pooled estimates of per-act HIV acquisition probabilities between RAI and RVI from meta-analytic reviews, when also considering the fractions of sex acts which are RAI among VOICE and HVTN 907 participants. For example, assuming that 25% of sex acts of women practicing RAI are anal (i.e. 75% of acts are RVI), and that the per-act HIV acquisition probability is 5-fold higher during RAI than RVI, would lead to a 1.9-fold increase in HIV incidence rate among women practicing RAI compared to women only practicing RVI. This suggest that more data is still needed to better measure these HIV per-act acquisition probabilities and risks, especially within the context of recent scale-up of HIV prevention and treatment tools which can have different efficacies during RAI and RVI.

In conclusion, RAI was prevalent in the three cohorts analyzed for this study and its practice and frequency should be more systematically and more precisely recorded and reported by studies looking at sexual behaviors and HIV seroconversions, using standardized measures, culturally adapted study instruments and confidential interview methods. In particular, more data on the fraction of sex acts, or condomless sex acts, which are RAI (instead of the sole history of RAI practice) could help better understand RAI magnitude, its individual and contextual determinants, as well as its contribution to incident HIV as a public health problem [19].

### Electronic Supplementary Material

Below is the link to the electronic supplementary material.

Supplementary Material 1

## Data Availability

The data and material analyzed can be obtained following specific procedures.

## Code Availability

The code used for the presented analysis is available for review upon request.
