# Supplementary material for: The Association Between Heterosexual anal Intercourse and HIV Acquisition in Three Prospective Cohorts of Women
Source: AIDS Behav. 2023 Jul 1;27(12):4010–21. doi: 10.1007/s10461-023-04115-y (PMC10598156; doi:10.1007/s10461-023-04115-y)
Supplement: Supplementary file 1 — Supplementary Material 1 [file 10461_2023_4115_MOESM1_ESM.pdf]

The association between heterosexual anal intercourse and HIV acquisition in three prospective cohorts of women: supplementary material

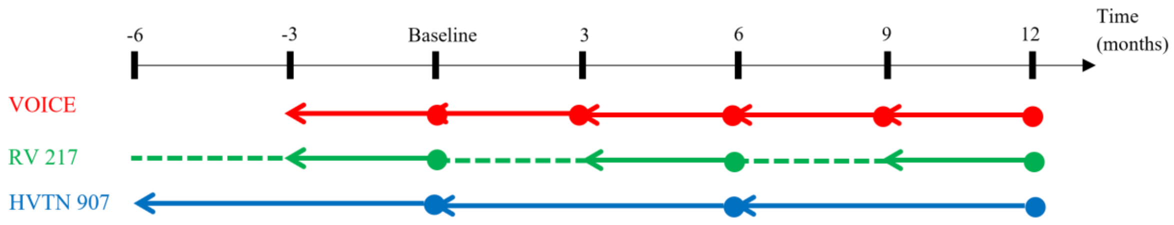

Figure S1. Reporting of RAI prevalence data during VOICE (red), RV 217 (green), and HVTN 907 (blue) longitudinal surveys. Each dot represents the time of the baseline or follow-up behavioural survey, whereas plain arrows represent the recall periods over which RAI prevalence is reported. The green dashed line represents periods of RV 217 survey which were not covered by the behavioural questionnaire, and our analysis assumes that the participants' report of RAI practice over their past six months was the same as over the past 3 months.

Table S1: Summary of characteristics of the study participants at baseline

	VOICE	RV 217	HVTN-907
Country	South Africa: 81%<br>Uganda: 6%<br>Zimbabwe: 13%	Kenya: 60%<br>Uganda: 40%	Dominican Republic: 32%<br>Haiti: 44%<br>Puerto Rico: 24%
Trial arm	Intervention: 60%<br>Placebo: 40%	NA	NA
Age	<25 years: 51%<br>≥25 years: 49%	<25 years: 62%<br>≥25 years: 38%	<25 years: 46%<br>≥25 years: 54%
Sex work	Yes: 6%[a]<br>No: 94%	Yes: 82%[b]<br>No: 18%	Yes: 100%[c]<br>No: 0%
Injection drug use	NA	Ever: 8%, including current: 2%	In the past 6 months: 3%<br>Not in the past 6 months: 97%
Number of partners	0: 2%[d]<br>1: 77%<br>2: 16%<br>3+: 5%	0: 7% [e]<br>1-2: 37%<br>3-10: 32%<br>10+: 24%	0-10: 15% [f]<br>11-100: 22%<br>101-1000: 51%<br>1000+: 12%
Number of sex acts	0: 28%[g]<br>1: 16%<br>2: 20%<br>3: 16%<br>4+: 20%	0-5: 7% [h]<br>6-20: 30%<br>21-90: 55%<br>91+: 8%	NA
Condom use at last RVI	Among RVI-only women: 71%<br>Among RAI+ women: 73%	At last intercourse (not reported if RAI or RVI)<br>a) with a steady partner:<br>RVI-only women: 45%<br>RAI+ women: 43%<br>b) with a casual partner:<br>RVI-only: 68%<br>RAI+: 48%<br>c) with a client:<br>RVI-only: 72%<br>RAI+: 53%<br>d) with another[i] type of partner:<br>RVI-only: 63%<br>RAI+: 49%	Among RVI-only women: 79%<br>Among RAI+ women: 73% [j]
Condom use at last RAI	69%	With a steady partner: 39%<br>With a casual partner: 43%<br>With a client: 47%<br>With another[i] type of partner: 47%	Among RAI+ women: 66%[k]

[a] Defined as having received money or good in exchange for sex in the past year
[b] Defined as having had a commercial partner in the past 3 months
[c] Defined as having received money or good in exchange for sex in the past 6 months
[d] Defined in the past 3 months
[e] Defined as the number of partners they had condomless sex with in the past 3 months (“0 partners” include those not skipping the question)
[f] Defined as the number of clients seen in the past 6 months
[g] Defined in the past week
[h] Defined in the past 3 months
[i] “other” partners include boss/work supervisor, teacher, other authority figure and relative other than spouse, student, employee/work subordinate, or rapist.
[j] Calculated as the fraction of commercial vaginal sex acts over the past 6 months involving condoms
[k] Calculated as the fraction of commercial anal sex acts over the past 6 months involving condoms

Table S2a: Summary of characteristics of the VOICE study participants at baseline stratified by reported RAI practice

	Participants reporting both anal and vaginal sex in last 3 months n (%)	Participants only reporting vaginal sex in last 3 months n (%)	p value[a]
Country			<0.001
South Africa	781 (92)	3171 (79)	
Uganda	24 (3)	292 (7)	
Zimbabwe	47 (5)	579 (14)	
Trial arm			0.74
Intervention	505 (59)	2423 (60)	
Placebo	347 (41)	1619 (40)	
Age			0.001
<25 years	474 (55)	2003 (50)	
≥25 years	378 (45)	2039 (50)	
Sex work in prior year			0.05
Yes	64 (8)	230 (6)	
No	788 (92)	3811 (94)	
Number of partners in past 3 months			0.001
0	15 (2)	40 (1)	
1	622 (73)	3160 (78)	
2	171 (20)	629 (16)	
3+	44 (5)	213 (5)	
Number of sex acts in past week			0.44
0	237 (28)	1146 (28)	
1	123 (14)	655 (16)	
2	180 (21)	780 (19)	
3	124 (15)	631 (16)	
4+	186 (22)	828 (21)	
Condom use at last RVI			0.36
Yes	568 (73)	2678 (71)	
No	214 (27)	1097 (29)	

[Ta] Calculated using Pearson χ^2 test

Table S2b: Summary of characteristics of the RV 217 study participants at baseline stratified by reported RAI practice

	**Participants reporting both anal and vaginal sex in last 3 months n (%)**	**Participants only reporting vaginal sex in last 3 months n (%)**	**p value[a]**
Country			0.02
Kenya	130 (54)	799 (62)	
Uganda	111 (46)	492 (38)	
Age			0.30
<25 years	156 (65)	787 (61)	
≥25 years	85 (35)	504 (39)	
Sex work in past 3 months			0.03
Yes	213 (88)	1067 (83)	
No	28 (92)	224 (17)	
Ever injected a drug			<0.001
Yes	33 (14)	82 (6)	
No	207 (86)	1205 (94)	
Number of sexual partners[b]			<0.001
0 or unknown	9 (4)	93 (7)	
1-2	53 (22)	512 (40)	
3-10	89 (37)	410 (32)	
11+	89 (37)	272 (21)	
Number of sex acts in past 3 months			0.04
0-5	12 (6)	80 (7)	
6-20	53 (24)	359 (31)	
22-90	126 (58)	633 (55)	
91+	25 (12)	82 (7)	
Condom use at last sex act[c]			<0.001
Yes	112 (50)	846 (70)	
No	114 (50)	364 (30)	

[a] Calculated using Pearson χ^2 test

[b] Reported as the total number of sexual partners with whom the participant had condomless sex in past 3 months

[c] Calculated as condom use at last sex act with client, and with a steady partner if the latter was not reported

Table S2c: Summary of characteristics of the HVTN 907 study participants at baseline stratified by reported RAI practice

	Participants reporting both anal and vaginal sex in last 6 months n (%)	Participants only reporting vaginal sex in last 6 months n (%)	p value[a]
Country			<0.001
Haiti	65 (27)	321 (50)	
Puerto Rico	105 (44)	109 (17)	
Dominican Republic	70 (29)	209 (33)	
Age			<0.001
<25 years	82 (34)	320 (50)	
≥25 years	158 (66)	319 (50)	
Sex work in past 6 months			N.A.
Yes	240 (100)	639 (100)	
No	0 (0)	0 (0)	
Ever injected a drug			0.71
Yes	9 (4)	19 (3)	
No	231 (96)	620 (97)	
Number of clients in the past 6 months			<0.001
0-10	64 (27)	69 (11)	
11-100	65 (27)	131 (20)	
101-1000	88 (37)	358 (56)	
1000+	23 (9)	81 (13)	
Fraction of commercial vaginal sex acts over the past 6 months involving condoms[b]			0.009
	73%	79%	

[a] Calculated using Pearson χ^2 test

[b] Calculated using Wilcoxon signed-rank test

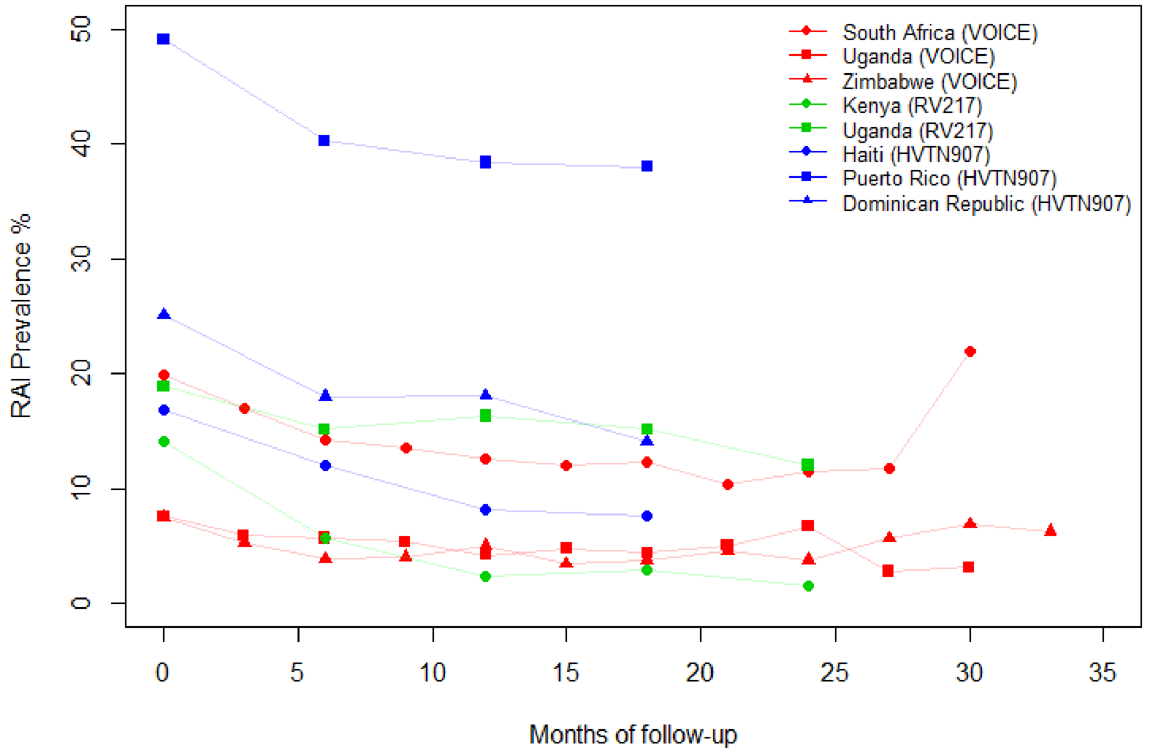

Figure S2. Cross-sectional RAI prevalence for the three longitudinal studies under consideration (VOICE, RV 217, and HVTN 907), by participants country of residence. Differences between RAI prevalence in Uganda as reported by the women from VOICE and RV 217 (red and green lines) are partly due to the fact that the RV 217 study enrolled women at higher risk of infection.

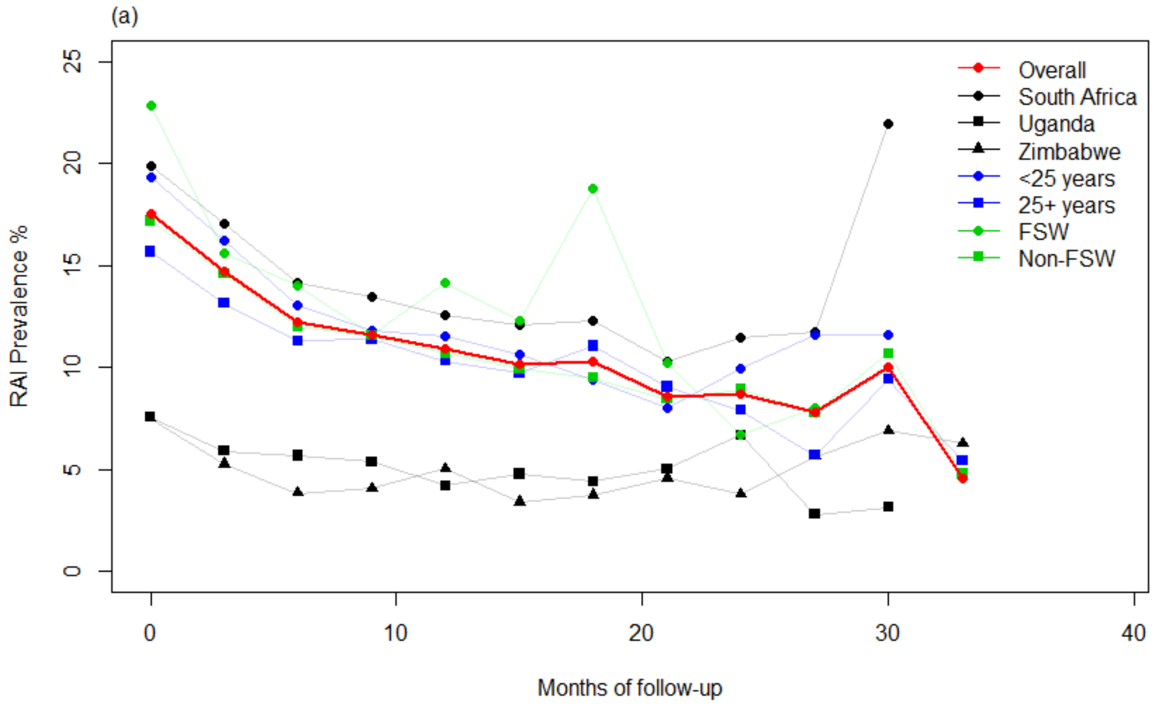

(a)
RAI Prevalence %
Months of follow-up
Overall
South Africa
Uganda
Zimbabwe
<25 years
25+ years
FSW
Non-FSW

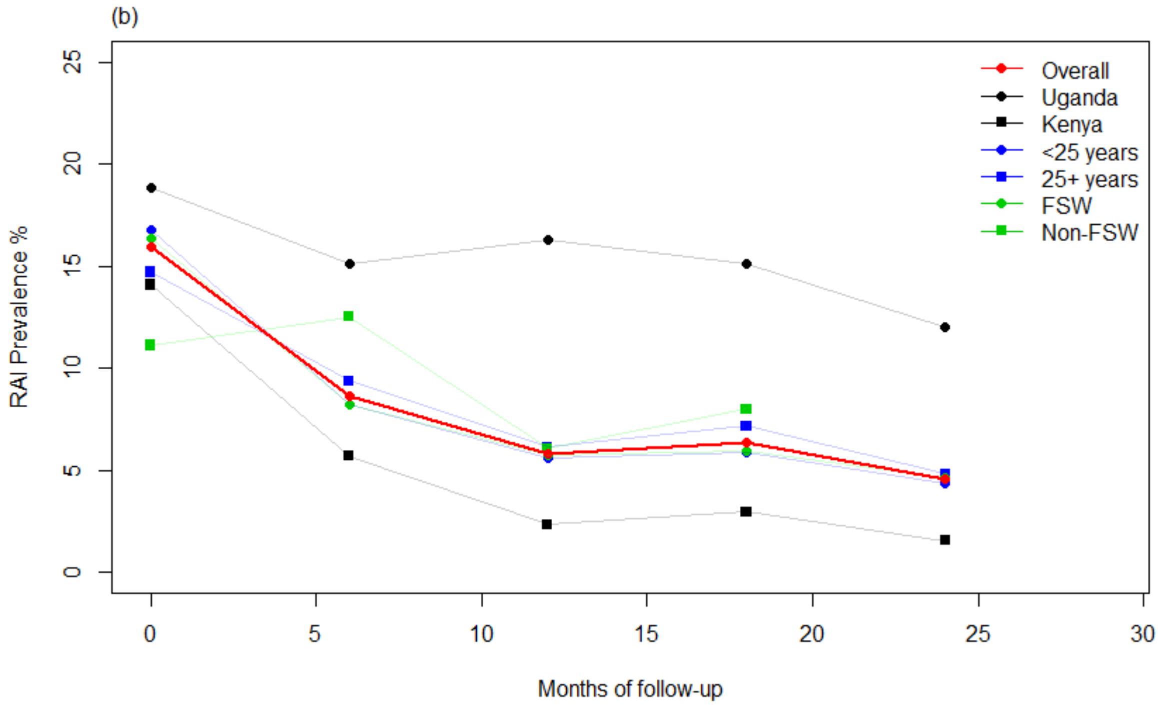

(b)
RAI Prevalence %
Months of follow-up
Overall
Uganda
Kenya
<25 years
25+ years
FSW
Non-FSW

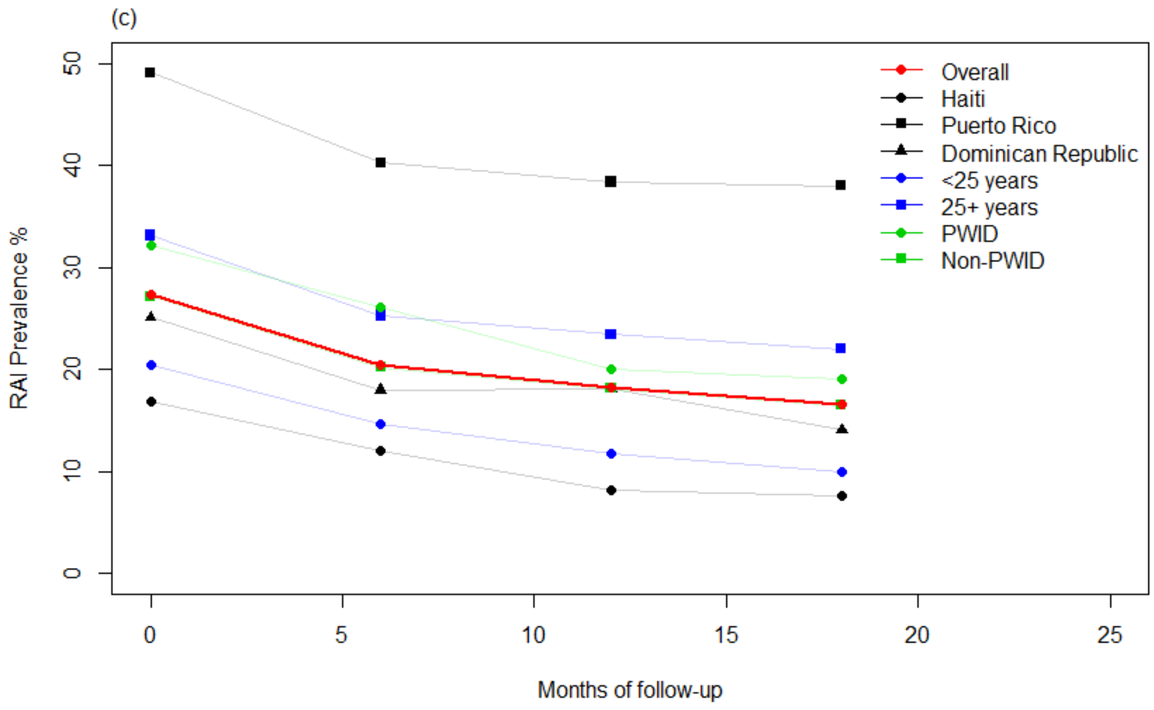

Figure S3: Cross-sectional RAI prevalence during a) MTN-003 VOICE, b) RV 217, c) HVTN 907 studies, stratified by individual characteristics.

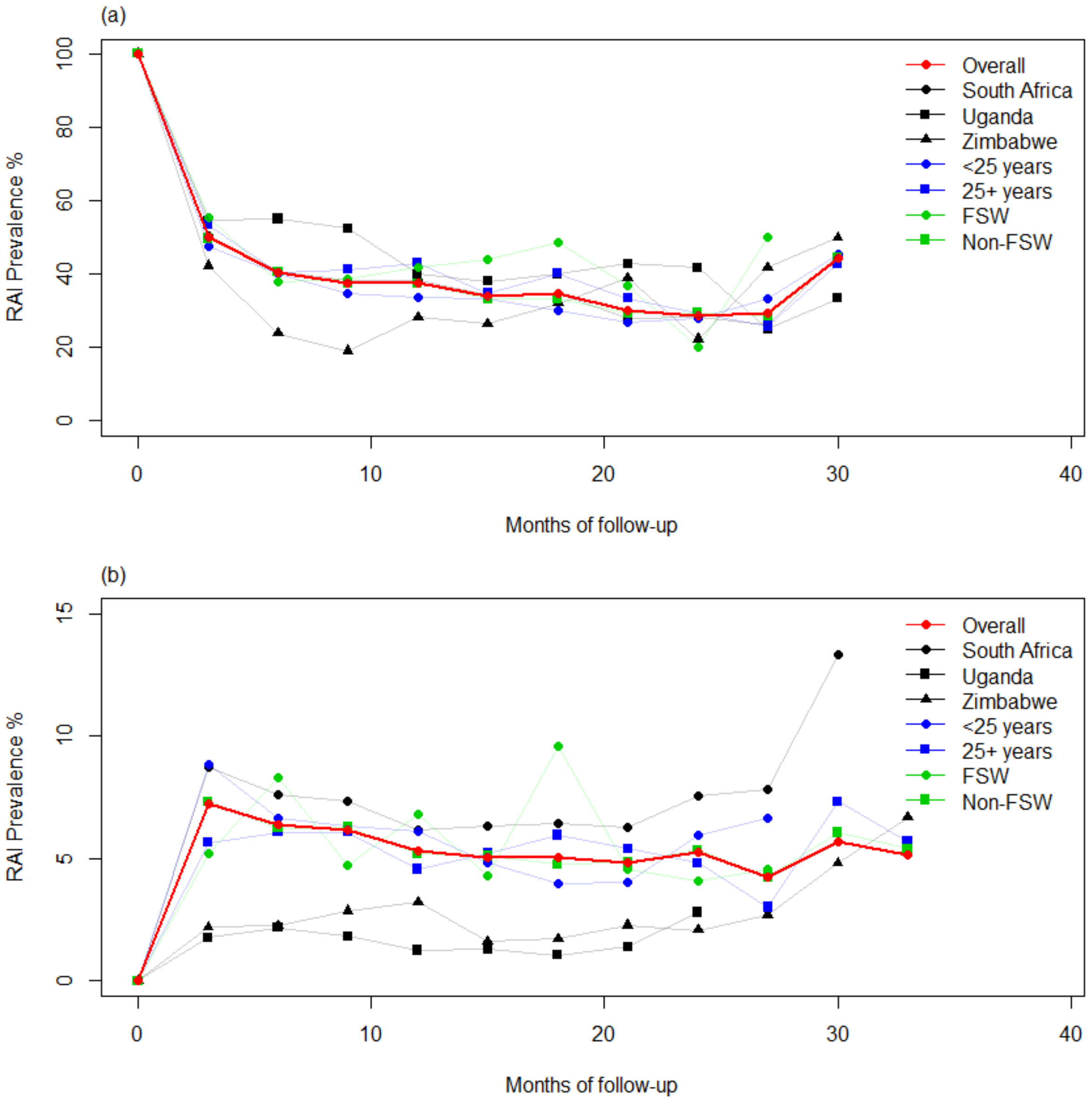

Figure S4: Cross-sectional RAI prevalence during VOICE among (a) RAI+ women and (b) RVI-only women at baseline, stratified by individual characteristics.

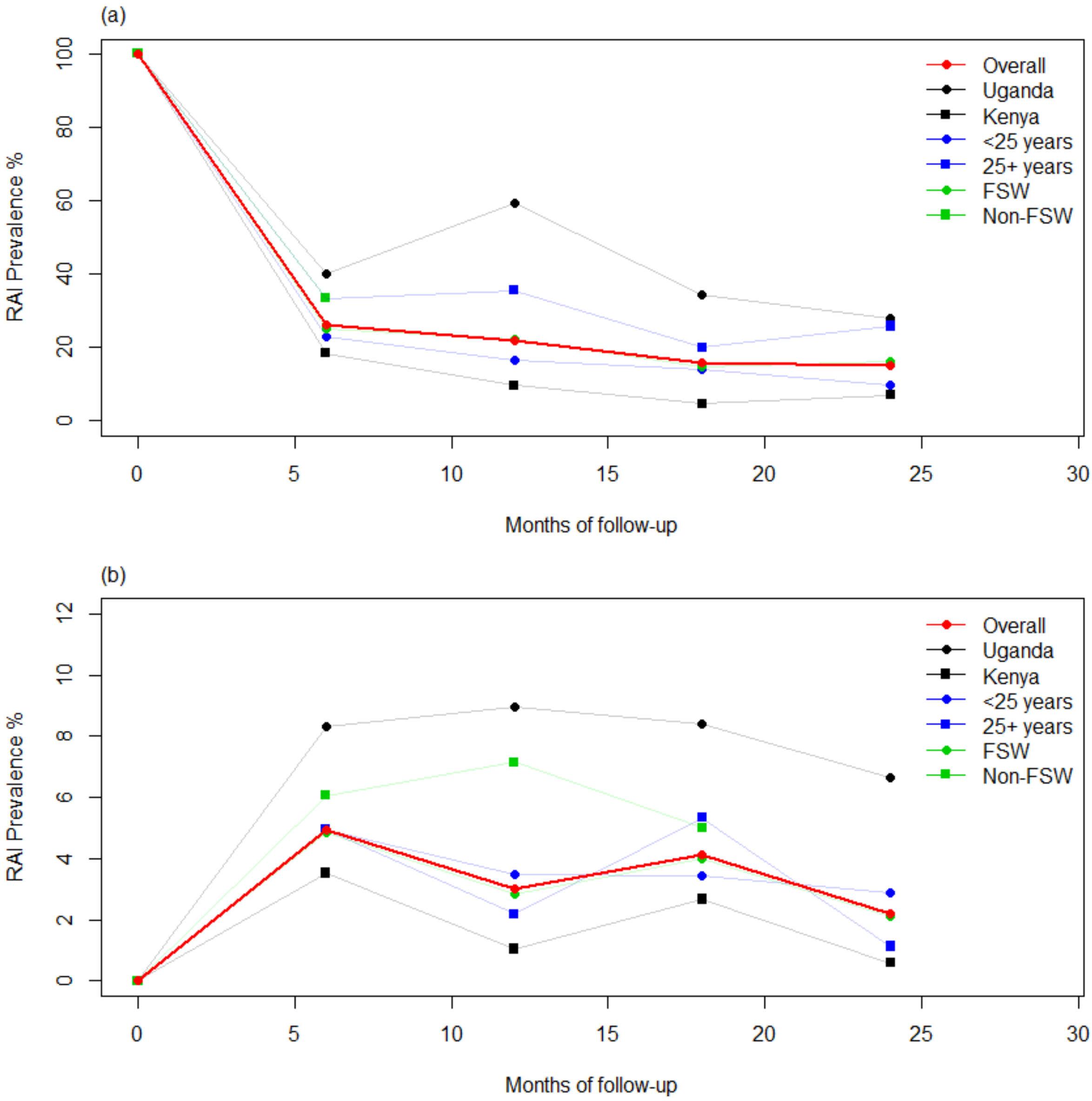

Figure S5: Cross-sectional RAI prevalence during RV 217 among (a) RAI+ women and (b) RVI-only women at baseline, stratified by individual characteristics.

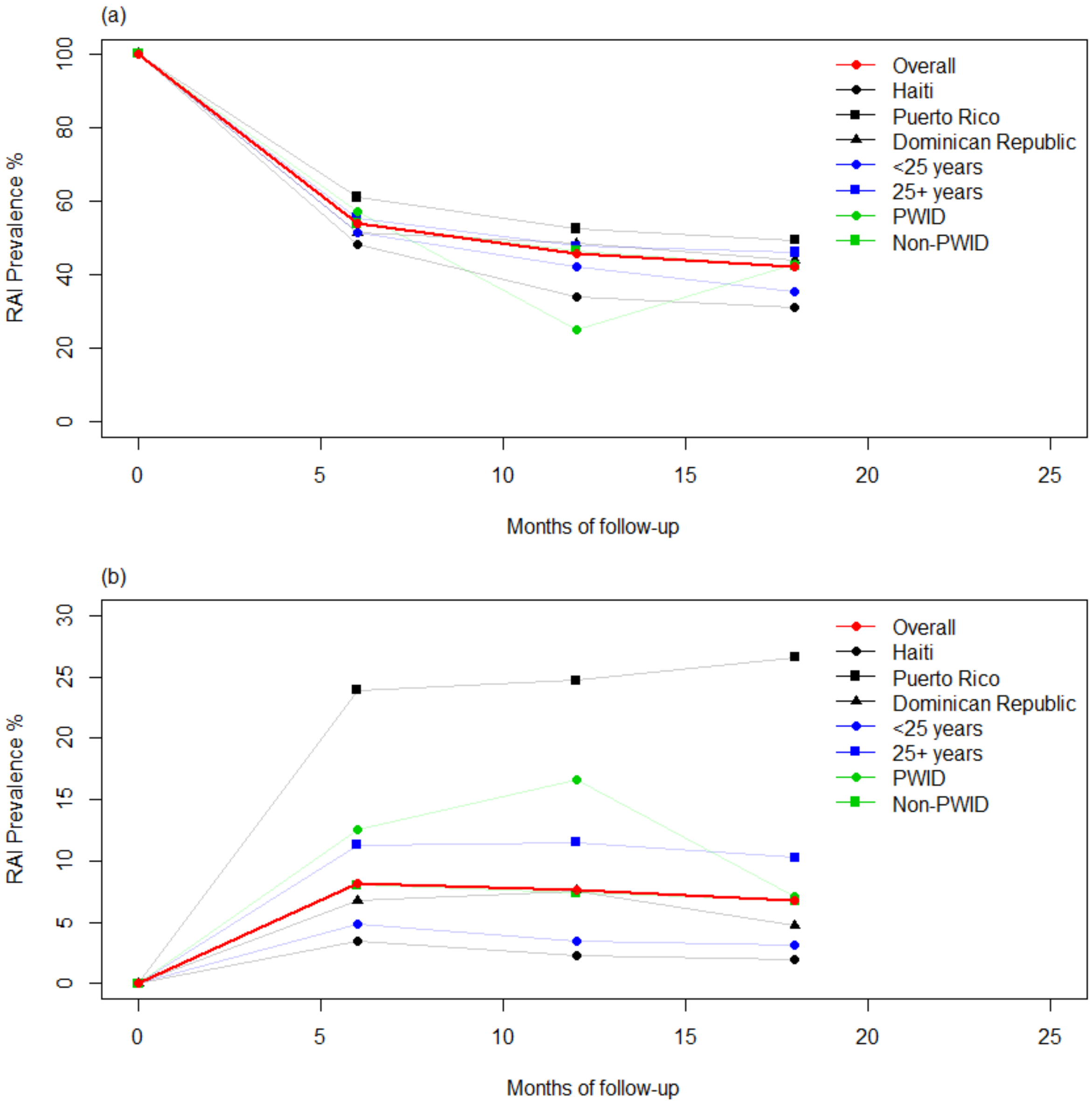

Figure S6: Cross-sectional RAI prevalence during HVTN 907 among (a) RAI+ women and (b) RVI-only women at baseline, stratified by individual characteristics.

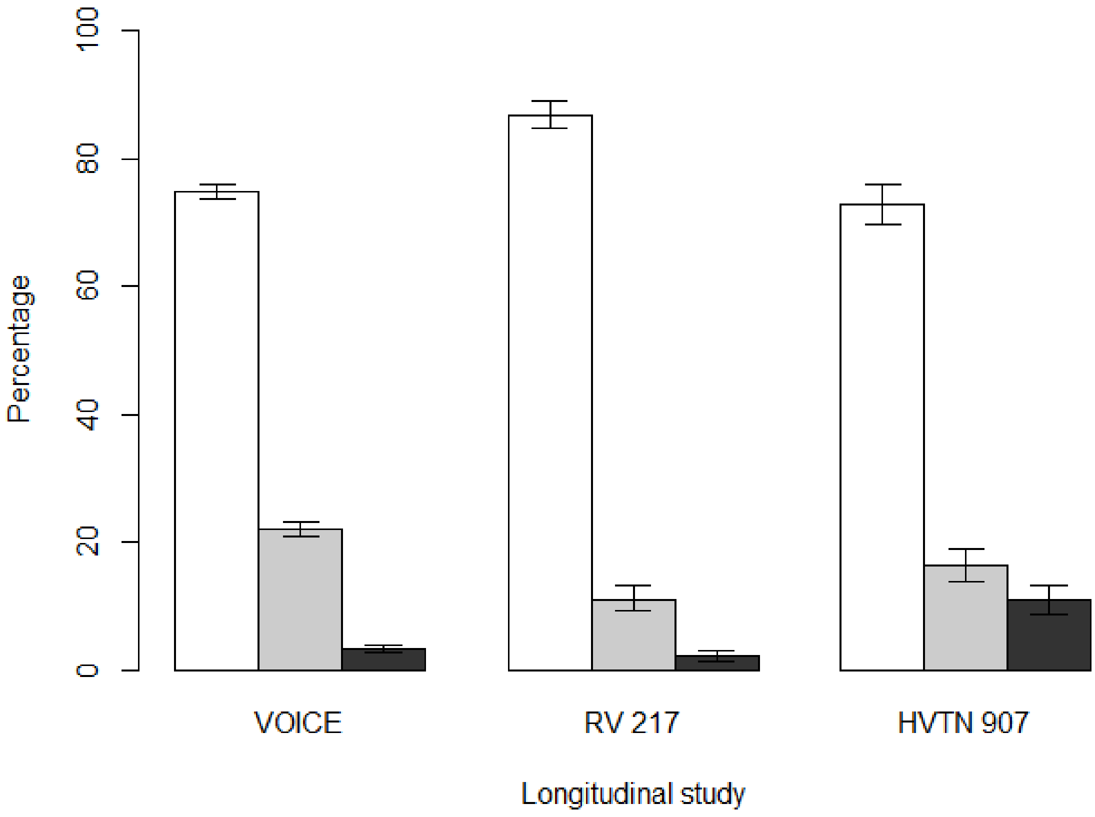

Figure S7. Persistence of RAI practice: Proportion of individuals of each study that never (white bars), sometimes (grey bars), or always (dark bars) reported RAI during follow-up. Errors bars represent 95% confidence interval of estimate.

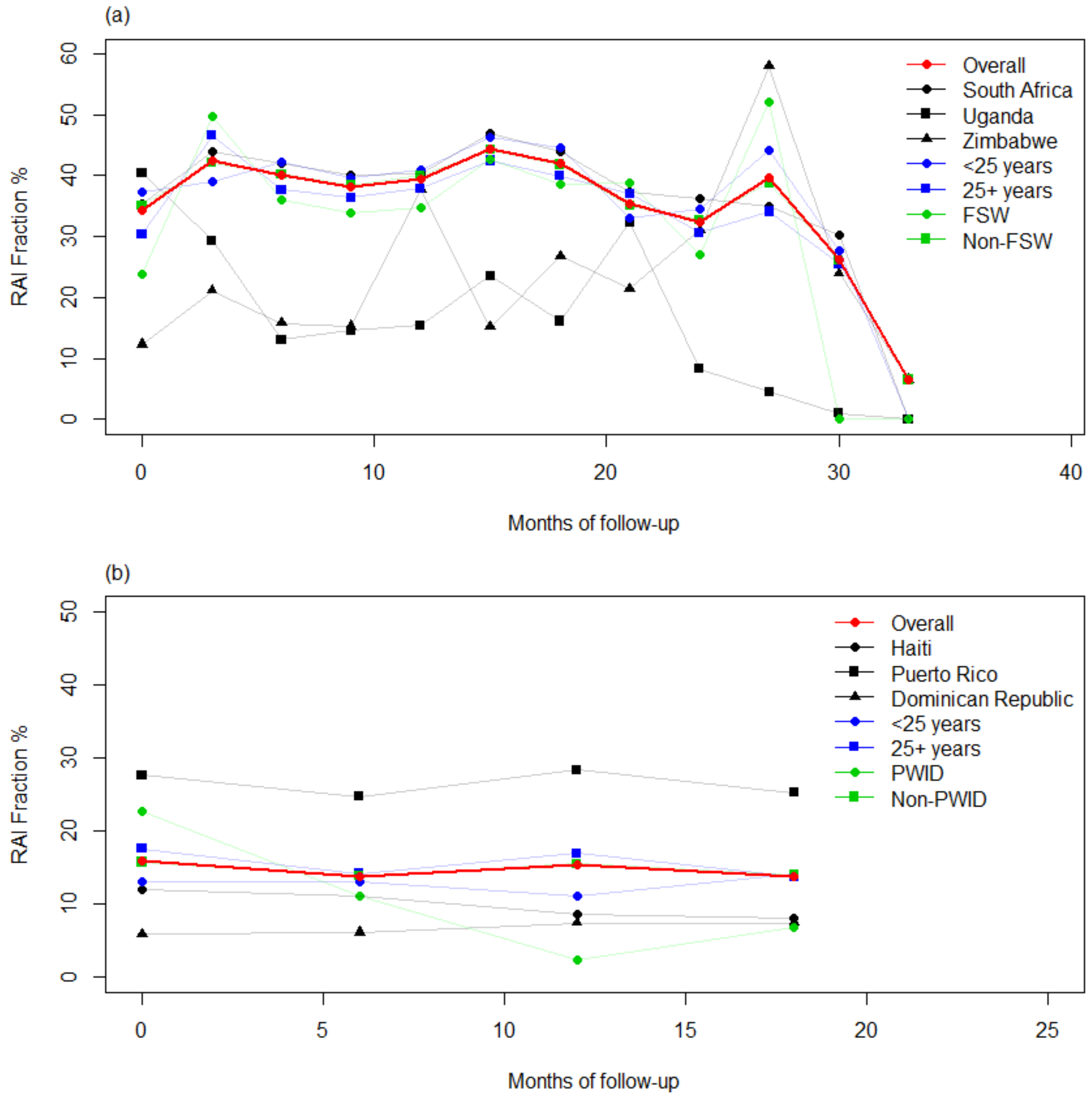

Figure S8: Cross-sectional RAI fraction (% sex acts that are anal among individuals reporting RAI) during VOICE and HVTN 907, calculated among subpopulations.

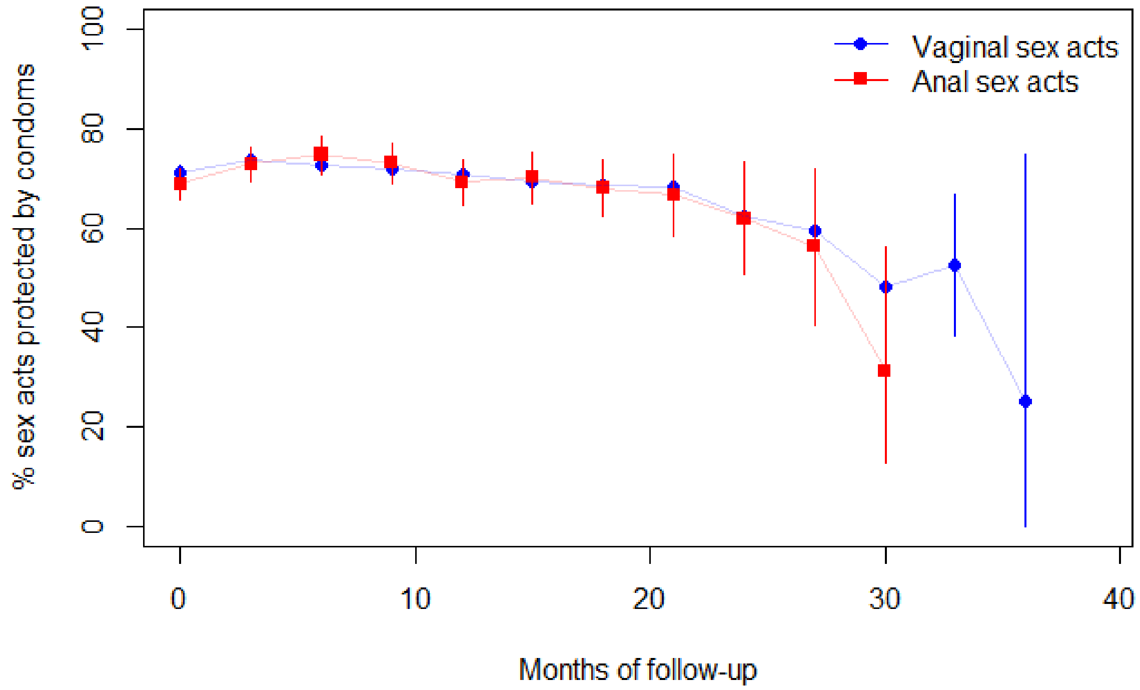

Figure S9: Cross-sectional proportion of last vaginal (blue) and anal (red) sex acts during which condoms were used, as reported by VOICE participants. Errors bars represent 95% confidence interval of estimate.

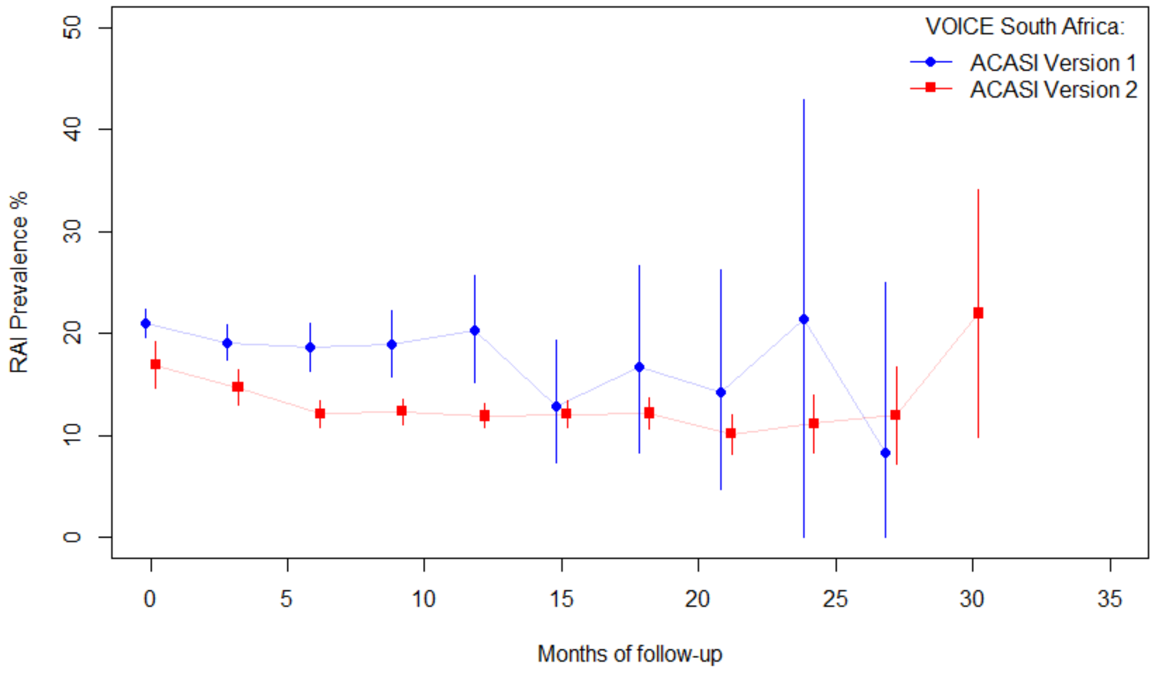

Figure S10: Cross-sectional RAI prevalence during VOICE by ACASI questionnaire version, reported by South African participants. Errors bars represent 95% confidence interval of estimate.

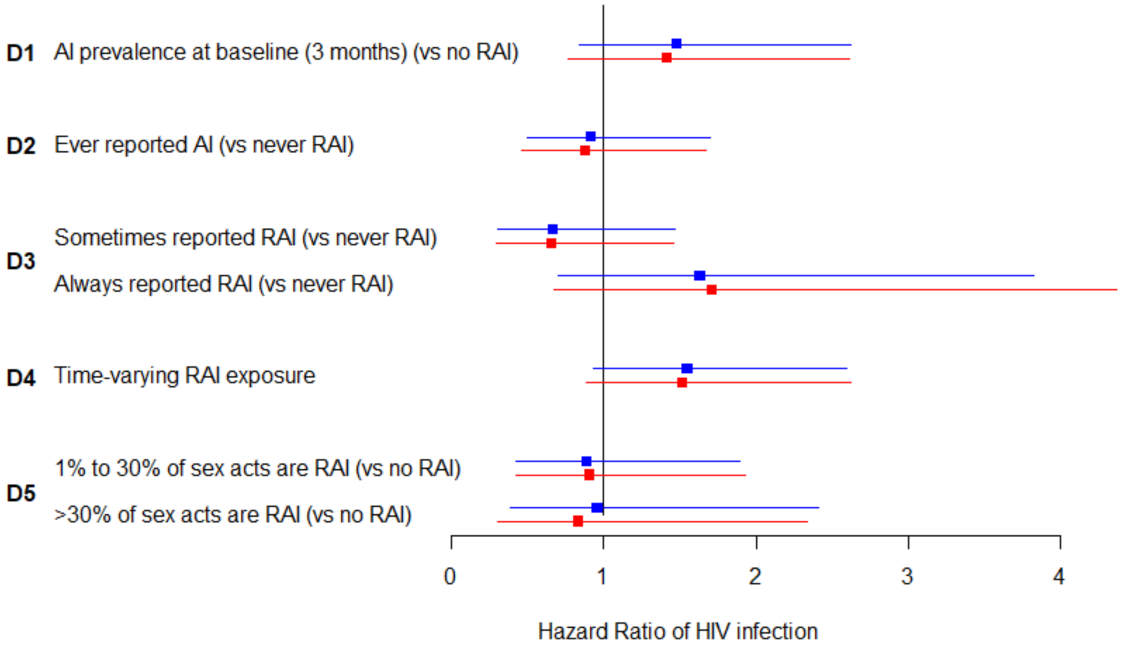

Figure S11: Increase in HIV incidence among women practicing RAI during VOICE, Unadjusted (blue) and adjusted (red) estimates of the association between different exposures of RAI and HIV incidence during VOICE, only among those who used the second version of the ACASI questionnaire (23% of participants). Confounding variables include country, age, trial arm, sex work in the prior year, number of partners, and condom use at last vaginal sex. Errors bars represent 95% confidence interval of estimate.

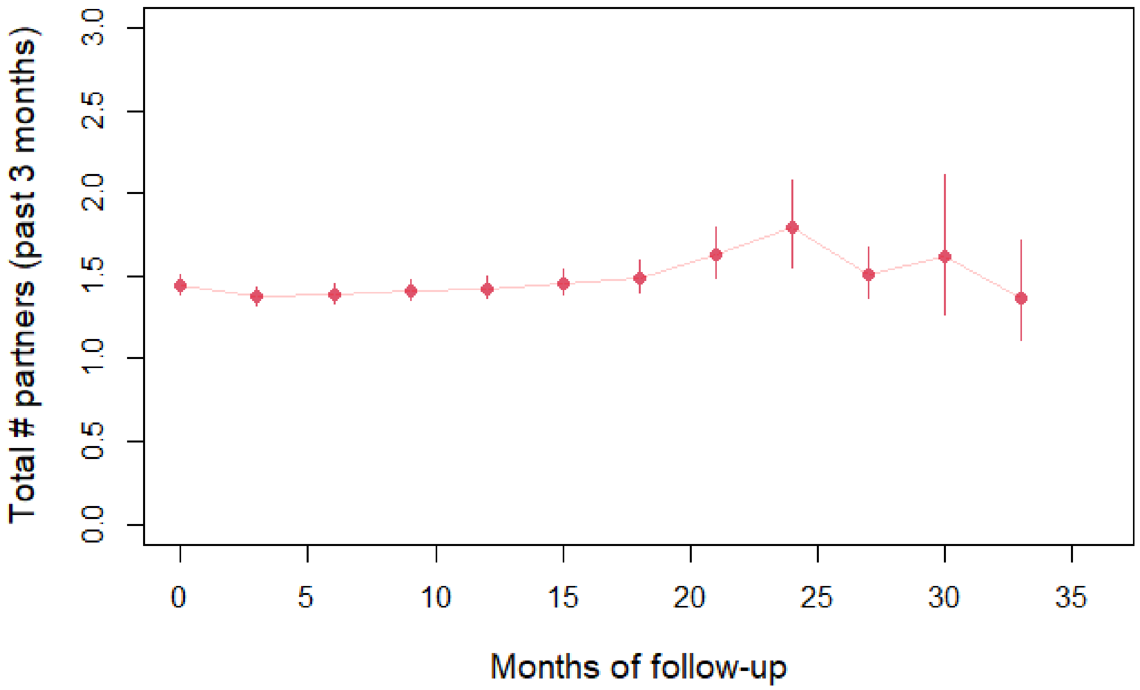

Figure S12: Cross-sectional total number of partners over the past 3 months reported by VOICE participants. Errors bars represent 95% confidence interval of estimate.
